# Bistable Expression of a Toxin-Antitoxin System Located in a Cryptic Prophage of Escherichia coli O157:H7

**DOI:** 10.1128/mBio.02947-21

**Published:** 2021-11-30

**Authors:** Dukas Jurėnas, Nathan Fraikin, Frédéric Goormaghtigh, Pieter De Bruyn, Alexandra Vandervelde, Safia Zedek, Thomas Jové, Daniel Charlier, Remy Loris, Laurence Van Melderen

**Affiliations:** a Cellular and Molecular Microbiology, Faculté des Sciences, Université Libre de Bruxelles (ULB), Gosselies, Belgium; b Structural Biology Brussels, Department of Bioengineering Sciences, Vrije Universiteit Brussel (VUB), Brussels, Belgium; c Molecular Recognition Unit, Center for Structural Biology, Vlaams Instituut voor Biotechnologie (VIB), Brussels, Belgium; d INSERM, CHU Limoges, RESINFIT, U1092, University of Limoges, Limoges, France; e Research Group of Microbiology, Department of Bioengineering Sciences, Vrije Universiteit Brussel (VUB), Brussels, Belgium; Racah Institute of Physics and the Harvey, The Hebrew University of Jerusalem, Israel; Yale School of Medicine

**Keywords:** phenotypic heterogeneity, single-cell analysis, genome stabilization

## Abstract

Type II toxin-antitoxin (TA) systems are classically composed of two genes that encode a toxic protein and a cognate antitoxin protein. Both genes are organized in an operon whose expression is autoregulated at the level of transcription by the antitoxin-toxin complex, which binds operator DNA through the antitoxin’s DNA-binding domain. Here, we investigated the transcriptional regulation of a particular TA system located in the immunity region of a cryptic lambdoid prophage in the Escherichia coli O157:H7 EDL933 strain. This noncanonical *paaA2-parE2* TA operon contains a third gene, *paaR2*, that encodes a transcriptional regulator that was previously shown to control expression of the TA. We provide direct evidence that the PaaR2 is a transcriptional regulator which shares functional similarities to the lambda CI repressor. Expression of the *paaA2-parE2* TA operon is regulated by two other transcriptional regulators, YdaS and YdaT, encoded within the same region. We argue that YdaS and YdaT are analogous to lambda Cro and CII and that they do not constitute a TA system, as previously debated. We show that PaaR2 primarily represses the expression of YdaS and YdaT, which in turn controls the expression of *paaR2*-*paaA2-parE2* operon. Overall, our results show that the *paaA2-parE2* TA is embedded in an intricate lambdoid prophage-like regulation network. Using single-cell analysis, we observed that the entire locus exhibits bistability, which generates diversity of expression in the population. Moreover, we confirmed that *paaA2-parE2* is addictive and propose that it could limit genomic rearrangements within the immunity region of the CP-933P cryptic prophage.

## INTRODUCTION

Type II toxin-antitoxin (TA) systems are generally composed of two genes organized in an operon that code for two proteins, a toxin and its cognate antitoxin. Under steady-state conditions, these two proteins form a strong complex in which the activity of the toxin is impeded. Such complexes have the capacity to bind operator sites in TA promoters to tightly repress transcription (1–3). The regulatory activity is mainly exerted by antitoxins through their DNA-binding domain, with toxins acting as corepressors (4). The *paaR2-paaA2-parE2* TA system is located on the CP-933P cryptic prophage of the enterohemorrhagic Escherichia coli strain O157:H7 EDL933. Cryptic prophages are generally abundant in bacterial genomes and are major drivers of genetic plasticity (5). The O157:H7 strain contains up to 12 lambda-like prophages, although only one (Shiga-toxin-converting phage CP-933W) is able to produce phage particles (6, 7). The *paaR2-paaA2-parE2* TA system constitutes a particular system in which transcription regulation is orchestrated by a distinct third component (8). A homologue of this system is located on another cryptic prophage (CP-933M) in the same strain (8).

Previous expression studies have shown that the *paaR2-paaA2-parE2* genes are organized in an operon and that transcription is downregulated by the PaaR2 regulator (8). Although the precise target of the ParE2 toxin remains to be uncovered, this toxin is likely to interfere with topoisomerases, since it colocalizes with the nucleoid and its overexpression induces the SOS response (8). The PaaA2 antitoxin, which is devoid of any distinctive DNA-binding domain, forms a complex with the ParE2 toxin in a highly cooperative manner (9). Moreover, PaaA2 is unstable and is degraded by the ClpAP and ClpXP ATP-dependent proteases (8). This TA system defies the typical autoregulatory mechanism observed for classical type II TA systems that relies on modulation of the DNA-binding affinity of the antitoxin by the toxin (1, 2, 10). In canonical type II TAs, a toxin-to-antitoxin ratio in favor of the toxin would lead to an alleviation of the repression ensuring antitoxin neosynthesis. This mechanism, dubbed “conditional cooperativity,” is thought to buffer any disturbance of the toxin-to-antitoxin ratio, thus avoiding stochastic release of toxin molecules. How these regulations are translated in the case of a tripartite system remains unknown. Here, we uncover an intricate regulatory network controlling the expression of the *paaR2-paaA2-parE2* locus. We provide evidence that the PaaR2 regulator, despite lacking sequence similarity, is an analog of the lambda repressor CI. PaaR2 exerts strong repression on the neighboring operon transcribed from the opposite strand, which encodes the YdaS and YdaT proteins of unknown functions. We show that these two proteins do not constitute a TA system, as previously debated (11–15), but rather encode analogs of the lambda Cro and CII transcriptional regulators, respectively. YdaS and YdaT regulate transcription of the *paaR2-paaA2-parE2* locus, and in turn, PaaR2 impacts transcription of the *ydaST* locus. We demonstrate that expression from this CP-933P immunity region can exist in two mutually exclusive states, likely corresponding to the lytic and lysogenic programs of the prophage, and that these transcriptional states are stable and transmitted to the progeny. Interestingly, the stationary phase induces switching from the *ydaST* (lytic) transcriptional state to the *paaR2-paaA2-parE2* (lysogenic) state, thus generating cell-to-cell heterogeneity in the population. However, neither transcriptional state was correlated with the activation of the SOS response under steady-state conditions, suggesting that the ParE2 toxin is not released in either subpopulation. Therefore, transcriptional activation appears to be uncoupled from the release of the toxin and subsequent growth inhibition, as has been recently demonstrated for other chromosomal TA systems (16). In addition, we show that the CP-933P immunity region is addictive and discuss the potential role of this system in prophage maintenance.

## RESULTS

### Genetic context of the *paaR2*-*paaA2*-*parE2* locus.

**FIG 1:**
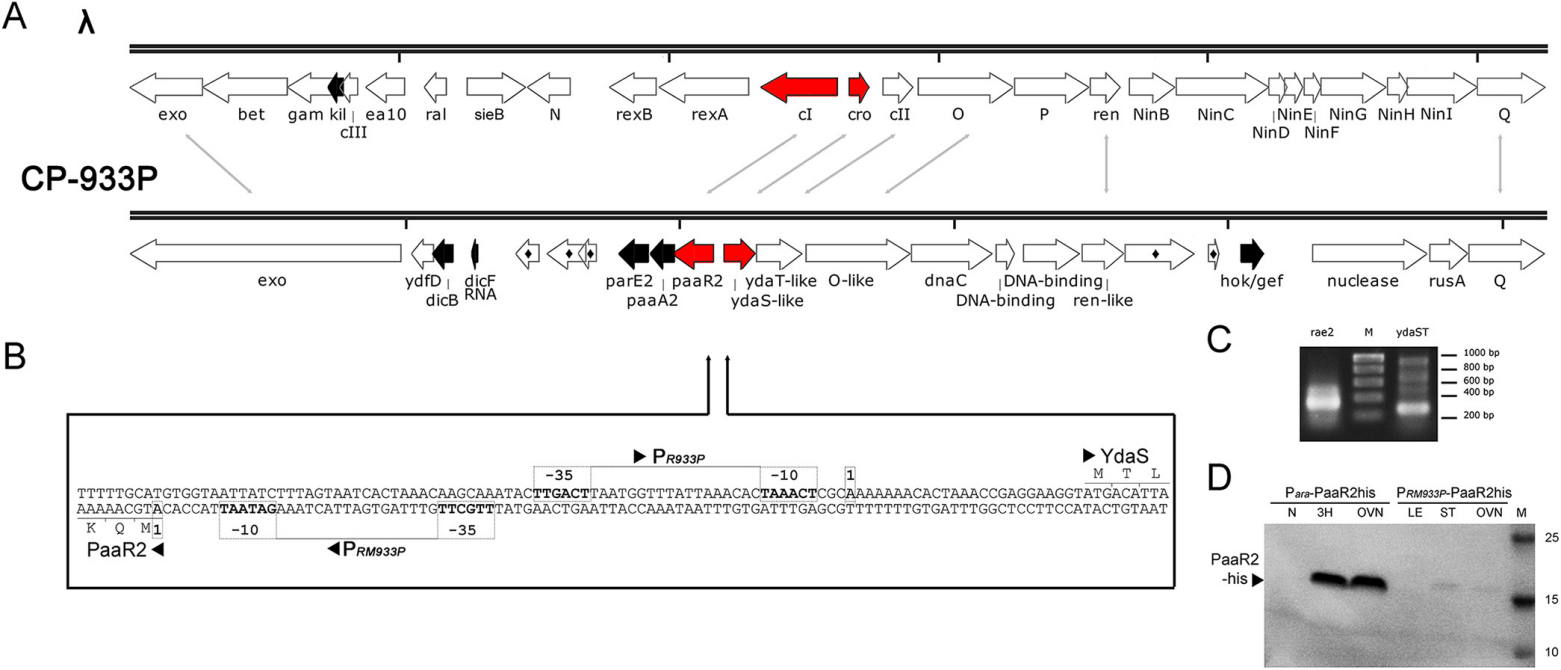
The CP-933P prophage shares common genetic structure with the lambda phage. (A) Comparison of the Escherichia coli K-12 lambda phage and the O157:H7 CP-933P prophage. Red arrows indicate the *paaR2* and *ydaS* open reading frames (ORFs). Black arrows indicate the *paaA2* and *parE2* ORFs and other host-killing ORFs. Diamonds indicate hypothetical proteins without predicted function. Scales are marked every 2,500 bp. (B) Focus on the intergenic region between the *paaR2* and *ydaS* genes. Predicted promoters P_R933P_ and P_RM933P_ are indicated, and −10 and −35 elements are boxed in gray. The transcription start sites determined by 5′ rapid amplification of cDNA ends (RACE) (C) are indicated as +1. (C) Transcription starts of *paaR2* and *ydaS* determined by 5′ RACE. The gel shows 5′ RACE reactions amplified by PCR after reverse transcription. (D) Leaderless translation of *paaR2* was assayed by expressing the *paaR2-His* gene under the control of the P*_araBAD_* in the pBAD24 vector and under the control of its own promoter P_RM933P_ by replacing the P*_araBAD_* promoter. DJ624 Δ*ara* cells containing the aforementioned constructs were grown overnight, and protein extracts were resolved by SDS-PAGE followed by Western blotting with an anti-His antibody. N, not induced; 3H, 3 h of induction with arabinose starting at an optical density at 600 nm (OD_600_) of 0.3; OVN, overnight induction; LE, late exponential phase; ST, stationary phase; M, molecular weight marker.

Comparison of the CP-933P prophage to the organization of the lambda prophage shows that the *paaR2-paaA2-parE2* (*RAE2*) locus is located in the immunity region, upstream of a homolog of the *o* gene (Fig. 1A). Given the transcriptional regulatory activity of PaaR2 (8) and the left orientation (according to lambda terminology) of the corresponding gene, we hypothesized that PaaR2 is a functional equivalent of the CI repressor. The lambda CI repressor regulates its own expression and ensures the maintenance of the lysogenic state by repressing the transcription of lytic genes (17). In addition to CI, lambda encodes the Cro and CII transcriptional regulators that participate in the lysis-lysogeny switch (reviewed in references 18–20). Cro represses the expression of CI and is essential for the lysogeny-lysis switch (21). On the other hand, CII is expressed during early infection (22). Upon accumulation, CII stimulates the expression of genes required to establish stable lysogens, among those the CI repressor (22). The *cro* and *cII* genes are located between the *cI* and *o* genes and are transcribed opposite to *cI*. Similarly, in CP-933P, two genes are located between the *paaR2* and *o* genes and are transcribed in the opposite direction to *paaR2*. These genes encode homologs of the YdaS (26% amino acid sequence identity) and the YdaT (31% sequence identity) proteins encoded by the E. coli K-12 Rac prophage. Further comparison of the *paaR2* genomic context with various lambdoid prophages using the FlaGs tool (23) revealed high levels of similarity between the *paaR2*-encoding region and lambdoid immunity regions (see Fig. S1 in the supplemental material). The YdaS-like protein from CP-933P (here referred to as YdaS) forms a homology cluster with Cro regulators from phages lambda, P22, and Phi80, but also with CII from lambda and CI from P22; YdaT-like protein from CP-933P (here referred to as YdaT) is homologous to CII from Phi80 (Fig. S1). Other similarities in genomic context include the presence of DNA replication initiation genes (e.g., *o*) in the 3′ region of *cII* or *ydaT* and division inhibitor-encoding genes (*kil* and/or *dicB*) in the 3′ region of *cI* or *paaR2* (Fig. S1). The YdaS protein family (COG4197) is reported as a putative transcriptional regulator possessing an HTH_XRE DNA-binding motif and belonging to the Cro superfamily (24). It has also been reported to be the antitoxin of the YdaT toxin (Pfam accession number DUF1019) or to be a toxin itself (12, 13, 25). Comparison of immunity regions in different lambdoid prophages suggest that the *paaR2* and *ydaS* genes in the related Sp12 cryptic prophage in the O157:H7 Sakai strain (identical to CP-933P) could encode functional analogs of lambda CI repressor and the Cro antirepressor, respectively (26). We propose that CP-933P-encoded PaaR2, YdaS, and YdaT are functional analogues to CI, Cro, and CII, respectively, and that the entire region comprising the *RAE2* and *ydaS-ydaT* operons constitutes the immunity region of CP-933P, given its similarity to the immunity regions of other lambdoid prophages in terms of organization, promoters, and regulation.

10.1128/mBio.02947-21.4 FIG S1 The CP-933P prophage shares a common outline with other lambdoid phages. FlaGs analysis of lambda, P22, Phi80, Qin, Rac, CP-933M, and CP-933P phages using five jackhammer iterations with a E value threshold of 0.05. Clusters of homologous genes are represented, each with a different color, while white-filled genes are not part of a cluster. Output (not to scale) was reprocessed to align homologous genes. Download FIG S1, JPG file, 0.5 MB. Copyright © 2021 Jurėnas et al. 2021 Jurėnas et al. https://creativecommons.org/licenses/by/4.0/ This content is distributed under the terms of the Creative Commons Attribution 4.0 International license.

### The *paaR2* regulator gene is translated in a leaderless manner, like *cI*.

Only a short intergenic region comprising 97 bp separates the predicted translation starts of *paaR2* and *ydaS*, which are in opposite directions (Fig. 1B). Based on sequence comparisons with the σ^70^ consensus sequence for E. coli promoters and with the corresponding lambda promoters P_R_ and P_RM_, potential promoters were predicted in this region (Table S1). Here, we refer to these promoters as P_RM933P_ (left orientation according to lambda nomenclature, leading to transcription of the *RAE2* locus) and P_R933P_ (right orientation, leading to transcription of the *ydaS-ydaT* locus) (Fig. 1B). To confirm the activity and the position of these promoters, 5′ rapid amplification of cDNA ends (RACE) experiments were performed. The detected +1 transcription start positions are in line with the predicted positions of P_R933P_ and P_RM933P_ promoters (Fig. 1B and C). The detected transcription start for P_RM933P_ matches the predicted translation start of the PaaR2 regulator protein, indicating that PaaR2 is translated in a leaderless manner (Fig. 1B). To confirm that the leaderless *paaR2* messenger is properly translated, we fused the *paaR2* open reading frame (ORF) to a C-terminal His tag and cloned under the control of the P*_araBAD_* promoter at the transcription initiation site of *araB* (27). The protein produced from this construct in the presence of arabinose is of similar molecular weight to that of the protein translated from its own promoter (Fig. 1D). The protein was purified using immobilized-metal affinity chromatography, and N-terminal protein sequencing established that the 5 N-terminal amino acids of the PaaR2-His protein are MQKKE, which corresponds to the translation from the transcript determined by 5′ RACE. This was further confirmed using mass spectrometry, where the intact mass measurement showed that PaaR2-His expressed from this construct has the expected mass of 15,081 Da. Importantly, only a few leaderless genes have been identified in E. coli, one of them being the lambda *cI* regulator gene, which was demonstrated to be expressed by translation initiation by the 70S ribosomes, therefore bypassing the need for initiation factors (28–31). Thus, the *paaR2* and *cI* genes share an unusual leaderless gene structure.

10.1128/mBio.02947-21.1 TABLE S1 Lambda prophage promoters in comparison to CP-933P prophage promoters and consensus promoter sequence (bold). Download Table S1, DOCX file, 0.02 MB. Copyright © 2021 Jurėnas et al. 2021 Jurėnas et al. https://creativecommons.org/licenses/by/4.0/ This content is distributed under the terms of the Creative Commons Attribution 4.0 International license.

### PaaR2 and YdaS bind specific operators in the intergenic region between the *paaR2-paaA2-parE2* and *ydaS-ydaT* operons.

**FIG 2:**
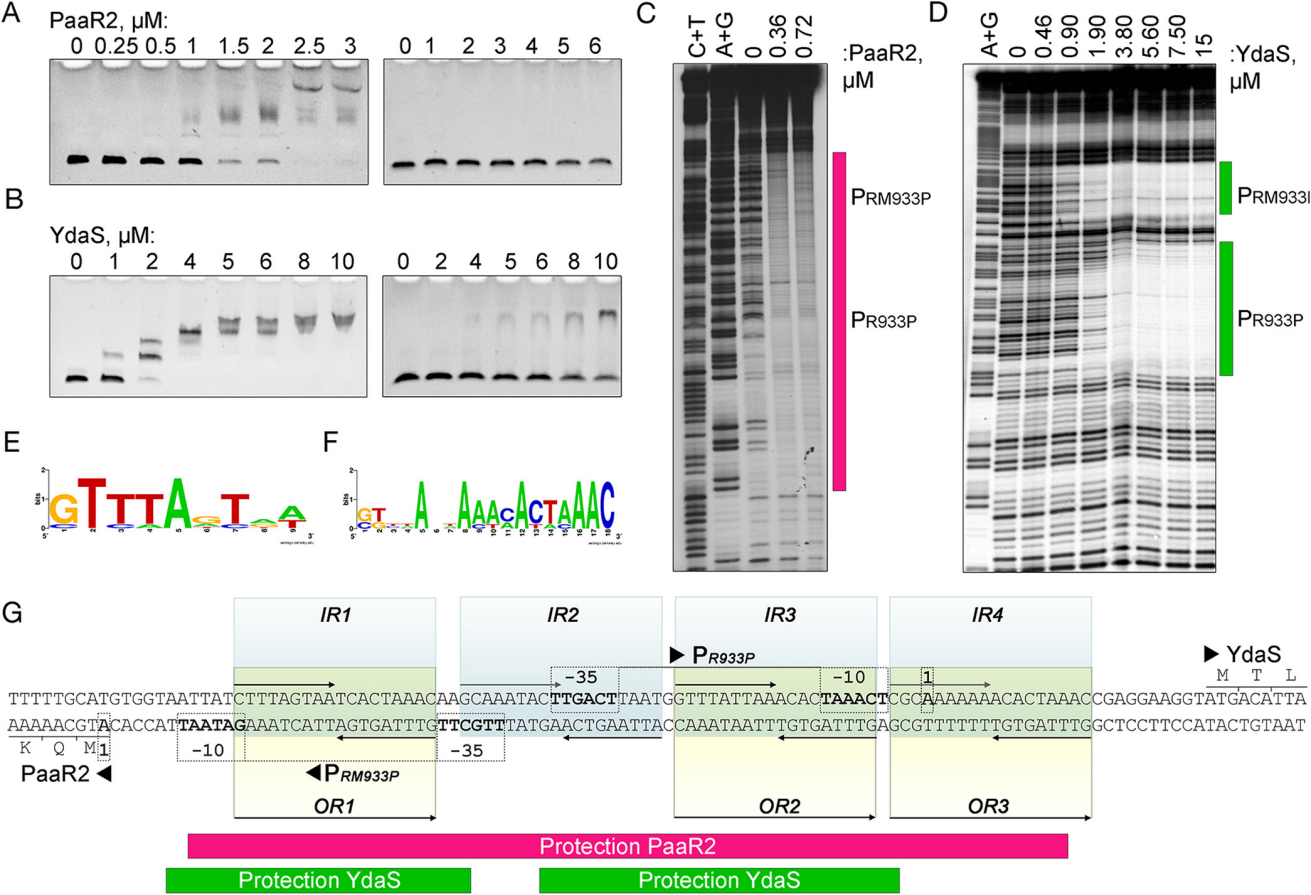
DNA binding of PaaR2 and YdaS to the intergenic region between the *RAE2* and *ydaST* loci. Electrophoretic mobility shift assays (EMSAs) and DNase I footprinting of PaaR2 (A, C) and YdaS (B, D) and the *paaR2-paaA2-parE2* and *ydaS-ydaT* intergenic region (left panels) or a random DNA sequence (right panels). Conservation and consensus of the inverted repeats (E) and operators (F) detected in the intergenic region. The consensus sequence was built from the alignment of the repeats located in the intergenic *ydaST-RAE2* region and a downstream *RAE2* region containing the P_L933P_ promoter (see Fig. S2 in the supplemental material). In total, 16 inverted repeat sequences and 6 operator sequences were aligned, and logo sequences were generated by WebLogo (https://weblogo.berkeley.edu/logo.cgi). (G) The regions protected by PaaR2 and YdaS in the intergenic region are marked in magenta and green, respectively.

The PaaR2 regulator has been shown to downregulate the transcription of the *RAE2* locus (8). The *ydaS* gene is located on the opposite strand (Fig. 1A and B) and encodes a protein harboring an HTH_Xre DNA-binding motif (24), indicating that this protein could also bind DNA. Electrophoretic mobility shift assays (EMSAs) were used to study the binding of PaaR2 and YdaS to the 97-bp intergenic region between the *RAE2* and *ydaS-ydaT* loci. EMSAs performed at increasing concentrations of YdaS and PaaR2 indicate that both proteins indeed bind this intergenic region at concentrations higher than 1 μM (Fig. 2A and B). Assays performed with a random DNA sequence at similar concentrations showed no binding, indicating that the binding to the intergenic region is specific (Fig. 2A and B). Binding of PaaR2 or YdaS to the intergenic region gave rise to multiple protein-DNA complexes (Fig. 2A and B), suggesting that this region contains several binding sites that are hierarchically bound by these proteins, resulting in decreased electrophoretic mobility. DNase I footprinting was used to delimit the regions bound by the two regulators within the 97-bp intergenic region. PaaR2 protects a continuous stretch of approximately 78 bp against DNase I cleavage (Fig. 2C), while YdaS covers a region of 65 bp, divided into two stretches of 27 and 32 bp, respectively (Fig. 2D). The binding sites of PaaR2 and YdaS detected by DNase I footprints both overlap the two promoters, P_RM933P_ and P_R933P_. YdaS binds the P_RM933P_ promoter at low concentrations and its own promoter, P_R933P_, at higher concentrations. This is comparable to what was shown for Cro binding, which first represses *cI* expression, and its own expression at higher concentration (32). The repression is established by subsequent binding to three operator sites, consisting of repeat sequences (32, 33). Analysis of the region protected by PaaR2 and YdaS in the DNase I footprinting assays allowed us to determine two types of repeats. Unlike lambda phage, which possess three 18-bp palindromic sequences (operators O_R_1, O_R_2, and O_R_3) (34), we detected four equally spaced 18-bp palindromes (inverted repeats [IR]) with the consensus sequence GTTTAGTAA (Fig. 2E). Two well-conserved palindromic sequences overlap the P_RM933P_ (IR1) and P_R933P_ (IR3) promoters, and two additional repeat sequences (IR2 and IR4) lack conservation on the left half-sites of the palindrome (Fig. 2E and G). Additionally, three pseudosymmetrical sequences (here called operators O_R1_ to O_R3_) overlap IR1, IR3, and IR4, as well as the P_RM933P_ and P_R933P_ promoters (Fig. 2F and G). Although the precise binding sequences of PaaR2 and YdaS remain to be determined, the DNase I footprint data indicate that they overlap. The striking balance of conservation between direct and inverted repeats suggest that both types of sequences could be engaged for regulation.

10.1128/mBio.02947-21.5 FIG S2 Predicted P_L933P_ promoter downstream of the *paaR2*-*paaA2*-*parE2* operon. Promoter elements are boxed; predicted inverted repeats and operators (described in Fig. 2E and F) are highlighted in blue and yellow, respectively. Download FIG S2, JPG file, 0.3 MB. Copyright © 2021 Jurėnas et al. 2021 Jurėnas et al. https://creativecommons.org/licenses/by/4.0/ This content is distributed under the terms of the Creative Commons Attribution 4.0 International license.

### Transcriptional regulations mediated by PaaR2 and YdaS are overlapping.

**FIG 3:**
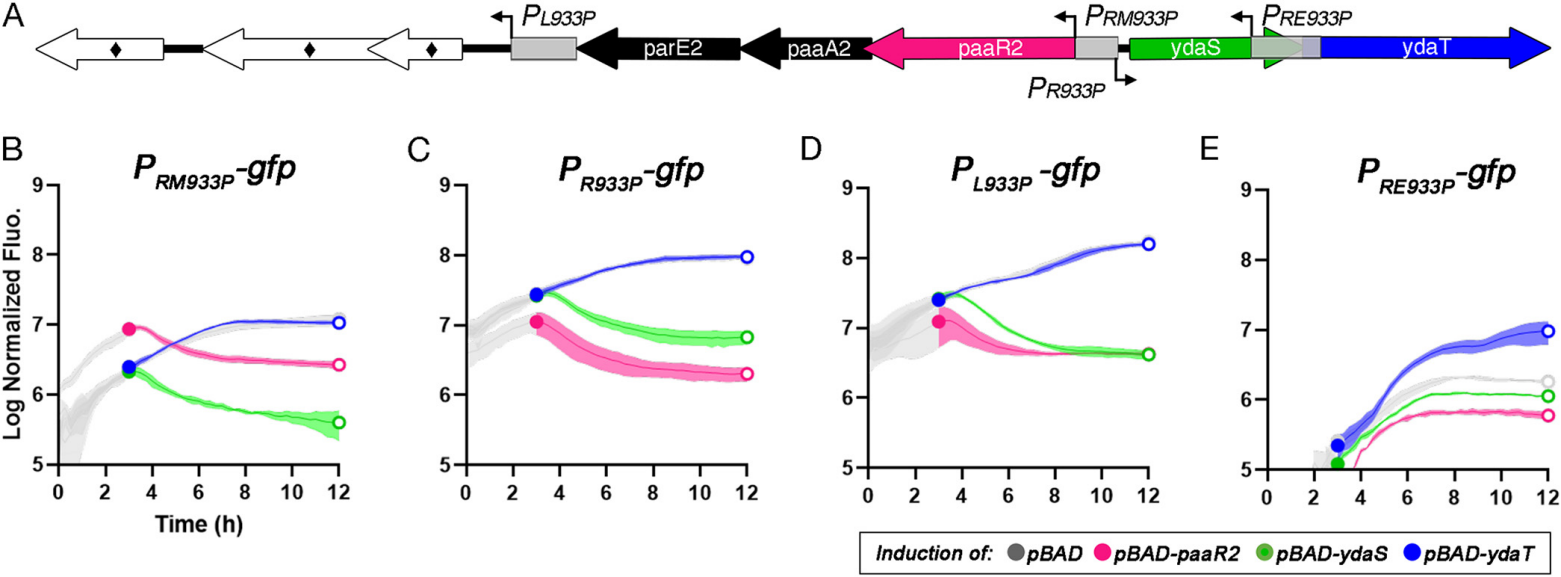
Lambdoid-like promoters of the CP-933P prophage are cross-regulated by PaaR2, YdaS, and YdaT. (A) Schematic representation of the promoters from CP-933P that were cloned upstream of the *gfp* gene and of the regulators (colored boxes) cloned downstream of the arabinose-inducible P*_araBAD_* promoter. P_RM933P_ (B), P_R933P_ (C), P_L933P_ (D), and P_RE933P_ (E) transcriptional activities were measured during 12 h of growth. Cells containing the pBAD24 vector (basal autofluorescence, gray), the pBAD-PaaR2 (magenta), the pBAD24-YdaS (green), or the pBAD24-YdaT (blue) plasmids were grown overnight, diluted to an OD_600_ of 0.02 (time zero) in M9 minimal medium with 0.2% glucose, grown until the early exponential phase, and induced with 0.2% arabinose after 3 h of growth. OD_600_ and fluorescence intensity were measured every 15 min. Curves represent mean values of three independent experiments; error bars indicate standard deviations.

The DNA sequences protected by both PaaR2 and YdaS in the DNase I footprinting experiments described above comprise the P_RM933P_ and P_R933P_ promoters, indicating that both proteins could regulate the expression of the *RAE2* and *ydaS-ydaT* loci (Fig. 2). To test this, transcriptional fusions of either the P_R933P_ or the P_RM933P_ promoter with the *gfp* gene were constructed, and green fluorescence was monitored under steady-state conditions or upon moderate production of PaaR2 or YdaS (Fig. 3A). The *paaR2* and *ydaS* genes were expressed from the pBAD24 vector by the addition of arabinose. Vectors carrying regulators and promoter reporters were cotransformed in the MG1655-derivative DJ624 Δ*ara* strain, which is devoid of the CP-933P prophage. After 3 h of growth, arabinose was added to induce the production of the PaaR2 or YdaS regulators, and fluorescence was monitored for 9 h (Fig. 3B and C). The basal activity of the P_RM933P_ promoter appears to be weaker than that of the P_R933P_ promoter. Expression of *paaR2* or *ydaS* resulted in the inhibition of *gfp* expression from both promoters, as visualized by a strong (up to 45-fold) decrease of fluorescence intensity per optical density at 600 nm (OD_600_) unit (Fig. 3B and C). This observation indicates that both promoters are repressed by PaaR2 and YdaS. Interestingly, during the first 3 h of growth prior to the expression of *paaR2* or *ydaS*, the basal fluorescence intensity of the strain containing the P_RM933P_ promoter reporter and the pBAD24-PaaR2 plasmid was around 4-fold higher than that of the strains containing the pBAD24 control vector or the pBAD24-YdaS plasmid. On the other hand, the basal green fluorescence intensity of the strain containing the P_R933P_ promoter reporter and the pBAD24-PaaR2 plasmid was 2.5-fold lower than that of the strains containing the pBAD24 control vector or the pBAD24-YdaS plasmid (Fig. 3B and C). These effects are likely due to the basal expression of *paaR2* or *ydaS* from the P*_araBAD_* promoter, even in the presence of glucose. Assuming this, the effects we observed under these conditions indicate that low levels of PaaR2 activate transcription from the P_RM933P_ promoter and therefore enhance the expression of the *RAE2* locus, while low levels of PaaR2 readily repress the P_R933P_ promoter (Fig. 3B and C). These observations are reminiscent of the lambda CI-mediated regulation, i.e., low levels of CI first fill O_R_1 and O_R_2 operator sites, cooperatively covering the P_R_ promoter, while the transcription from P_RM_ is activated by CI binding at the O_R_2 operator site, close to the −35 region of P_RM_ (35, 36). Meanwhile, Cro binds the same operator sites, but in the opposite order (32, 37). We also observed that the corresponding P_RM933P_ promoter was more strongly repressed by YdaS, while the P_R933P_ promoter was more strongly repressed by PaaR2 (Fig. 3B and C).

### PaaR2 and YdaS regulate an additional promoter in the CP-933P prophage.

In lambda, CI and Cro regulators also bind to the P_L_ promoter that is located upstream of the *cI-rexA-rexB* genes. Binding to distantly located O_L_ and O_R_ operators promotes octamerization of CI and enhances repression by forming a DNA loop (38–40). It is noteworthy that PaaR2, like CI, was found to octamerize in solution (41, 42). We thus wondered whether analogs of P_L_ and O_L_ exist in the CP-933P cryptic prophage. Upstream of the *RAE2* locus, we identified a putative promoter with −35 and −10 elements identical to those located in P_R933P_, as well as three operators (O_L1_, O_L2_, and O_L3_) similar to those identified around P_R933P_ and P_RM933P_. This putative promoter was named P_L933P_ by analogy to lambda (Fig. S2 and Table S2). The sequence comprising P_L933P_ was cloned upstream of the *gfp* gene in the same vector, as described above, and similar assays were performed (Fig. 3A). Green fluorescence intensity of the strains expressing *paaR2* or *ydaS* is markedly decreased upon arabinose addition (∼100-fold) compared to the control strain (Fig. 3D), indicating that both PaaR2 and YdaS negatively regulate the P_L933P_ promoter. A decrease in fluorescence intensity was observed during growth in the presence of glucose for the strain carrying the pBAD24-PaaR2 (Fig. 3D), indicating that low levels of PaaR2 repress the transcriptional activity of the P_L933P_ promoter. This effect is opposite to what we observed in the case of the P_RM933P_ promoter but is similar to the effect on the P_R933P_ promoter and consistent with the overlap of two types of conserved repeats within the P_L933P_ promoter sequence (Fig. 3B to D and Fig. S2). Note that no function can be assigned to the putative ORFs located downstream of P_L933P_ (Fig. 3A) based on sequence similarity or secondary structure predictions.

10.1128/mBio.02947-21.2 TABLE S2 Strains and plasmids used in this work. Download Table S2, DOCX file, 0.02 MB. Copyright © 2021 Jurėnas et al. 2021 Jurėnas et al. https://creativecommons.org/licenses/by/4.0/ This content is distributed under the terms of the Creative Commons Attribution 4.0 International license.

### YdaT provides alternative positive regulation of the *paaR2-paaA2-parE2* locus.

The YdaT protein from the Rac prophage is annotated as “toxin” and was predicted as a potential toxin by toxin-antitoxin search tools (14, 25, 43), although it has been previously argued that it is not a *bona fide* toxin (11, 15). We did not detect any toxic effect exerted by YdaT homologue from the CP-933P cryptic prophage (Fig. S3). Using the Dali server (44), structural similarity of the N terminus of YdaT homolog (PDB identifier 3C4R) to helix-turn-helix (HTH)-like DNA-binding domains in phage-borne transcriptional regulators (PDB identifiers 5A7L, 2MQK, and 3B7H) could be predicted, despite the lack of amino acid sequence similarity. Therefore, based on comparison to the genetic organization of the lambda immunity region, we tested whether YdaT from the CP-933P prophage could be a functional equivalent of the CII regulator. In lambda, CII plays an important role in lysogeny establishment by stimulating CI gene expression (45). CII binds to the P_RE_ promoter, which is located between the *cro* and *cII* genes and is otherwise silent (45). CII binding activates transcription from P_RE_, which results in the synthesis of an alternative transcript for *cI* expression (45–47). In our initial tests, we observed that expression of *ydaT* did not have any impact on the transcriptional activity of the P_RM933P_, P_RM933P_, or P_L933P_ promoters (Fig. 3B to D). We therefore searched for a remnant of P_RE_ in the CP-933P prophage between the *ydaS* and *ydaT* genes and identified a putative promoter, here called P_RE993P_ by analogy to lambda (Fig. 3E). The activity of this putative promoter was assayed as described above. The basal transcriptional activity of this promoter was below the detection level during growth in glucose. However, the fluorescence intensity was significantly increased upon *ydaT* expression, reaching fluorescence levels comparable to the basal fluorescence level of P_RM993P_ (Fig. 3B and E). These results suggest that P_RE993P_ is transcriptionally active in the presence of YdaT.

10.1128/mBio.02947-21.6 FIG S3 Growth inhibition by expressing genes from CP-933P immunity regions. Genes were cloned upstream of the P*_araBAD_* promoter in pBAD24 vector. Constructs were then transformed in the DJ624 Δ*ara* strain. Overnight cultures were serially diluted 10-fold (10^−2^ to 10^−8^ dilutions, shown from left to right), spotted on LB plates containing 0.2% glucose (repressive conditions) or 0.2% arabinose (inductive conditions), and grown overnight at 37°C. Download FIG S3, TIF file, 1.8 MB. Copyright © 2021 Jurėnas et al. 2021 Jurėnas et al. https://creativecommons.org/licenses/by/4.0/ This content is distributed under the terms of the Creative Commons Attribution 4.0 International license.

From these experiments, we conclude that PaaR2, YdaS, and YdaT are transcription regulators and confirm that the YdaS-YdaT pair is not a *bona fide* toxin-antitoxin module, as previously proposed (12, 13, 25). While prolonged overexpression of some of these regulators (in particular PaaR2 and YdaS) does cause growth defects (Fig. S3), a similar growth defect was also reported upon overexpression of some prophage regulators (15, 48). Despite this, the given genetic context and our evidence of transcription regulation suggest that YdaT is a transcriptional regulator that activates P_RE933P_, analogously to lambda CII.

### Bistability in the regulation of the *RAE2* and *ydaST* locus.

**FIG 4:**
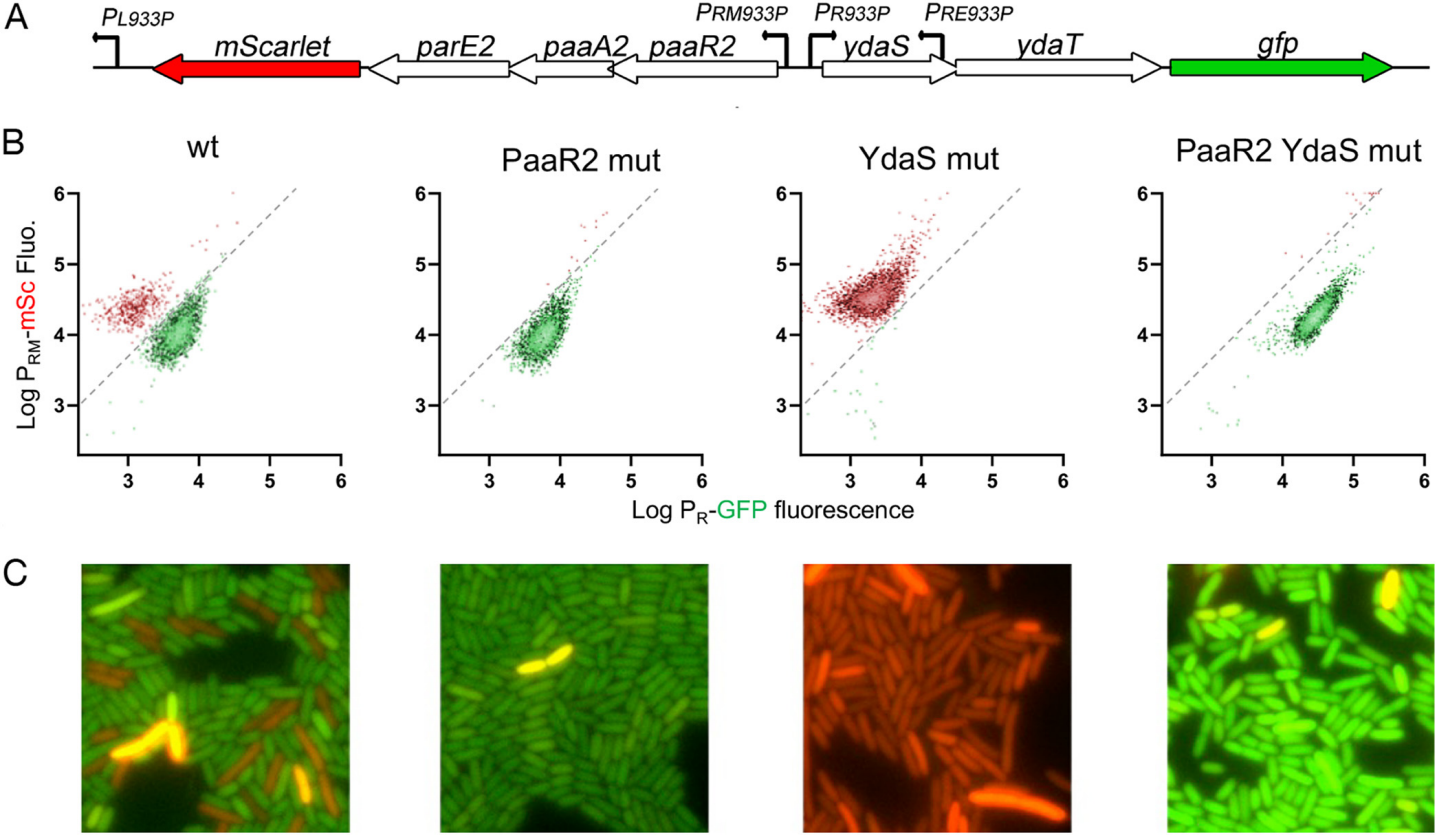
Transcriptional activity of the CP-933P immunity region. (A) Schematic representation of the pCP-933P vector containing the *paaR2-paaA2-parE2* and *ydaS-ydaT* loci in an operon with the mScarlet-I (mSc) or green fluorescent protein (GFP)-encoding genes, respectively (further indicated as wild type [wt]). (B) Fluorescence intensity distribution of 5,000 single FN042 (P*_sulA_-mTagBFP2*) cells transformed with wt and stop codon mutant pCP-933P constructs, measured by flow cytometry 6 h after overnight culture dilution in morpholinepropanesulfonic acid (MOPS) glucose medium. PaaR2^mut^, YdaS^mut^, and PaaR2^mut^ YdaS^mut^ are pCP-933P derivatives in which stop codon mutations are inserted in *paaR2*, *ydaS*, or both genes, respectively. (C) Fluorescence microscopy snapshots representative of each construct are shown below each plot. Gating is shown by dashed lines, with *ydaS*-on cells located to the right of the line and represented in green and *RAE2*-on cells located to the left of the line and represented in red.

To get insights into the contribution of the PaaR2 and YdaS regulators in the regulation of the expression of the *paaR2-paaA2-parE2* and *ydaS-ydaT* loci, a construct reporting the expression of both loci was engineered. This construct comprises the *ydaST-RAE2* immunity region with the *mScarlet-I* (*mSc*) gene inserted at the 3′ end of the *RAE2* operon and the *gfp* gene inserted at the 3′-end of the *ydaS-ydaT* operon (Fig. 4A). Red and green fluorescence of a strain transformed with this construct were followed using flow cytometry and fluorescence microscopy. In exponentially growing cells, we observed two populations with different fluorescent states (Fig. 4B and C). The majority of the population showed high levels of green fluorescent protein (GFP) fluorescence (indicative of high P_R_ transcriptional activity and high *ydaST* expression) coupled with low levels of mSc fluorescence (indicative of low P_RM_ transcriptional activity and low *RAE2* expression), while about a tenth of the cells exhibited high levels of mSc fluorescence and low levels of GFP fluorescence that were indistinguishable from autofluorescence (Fig. 4 to C and Fig. S4A). These subpopulations are referred to here as *RAE2*-on and *ydaST*-on, respectively. It is of note that few cells under each condition show bright mScarlet-I fluorescence. We suspect that this might be caused by random fluctuations of the copy number of the reporter-encoding plasmid.

**FIG 5:**
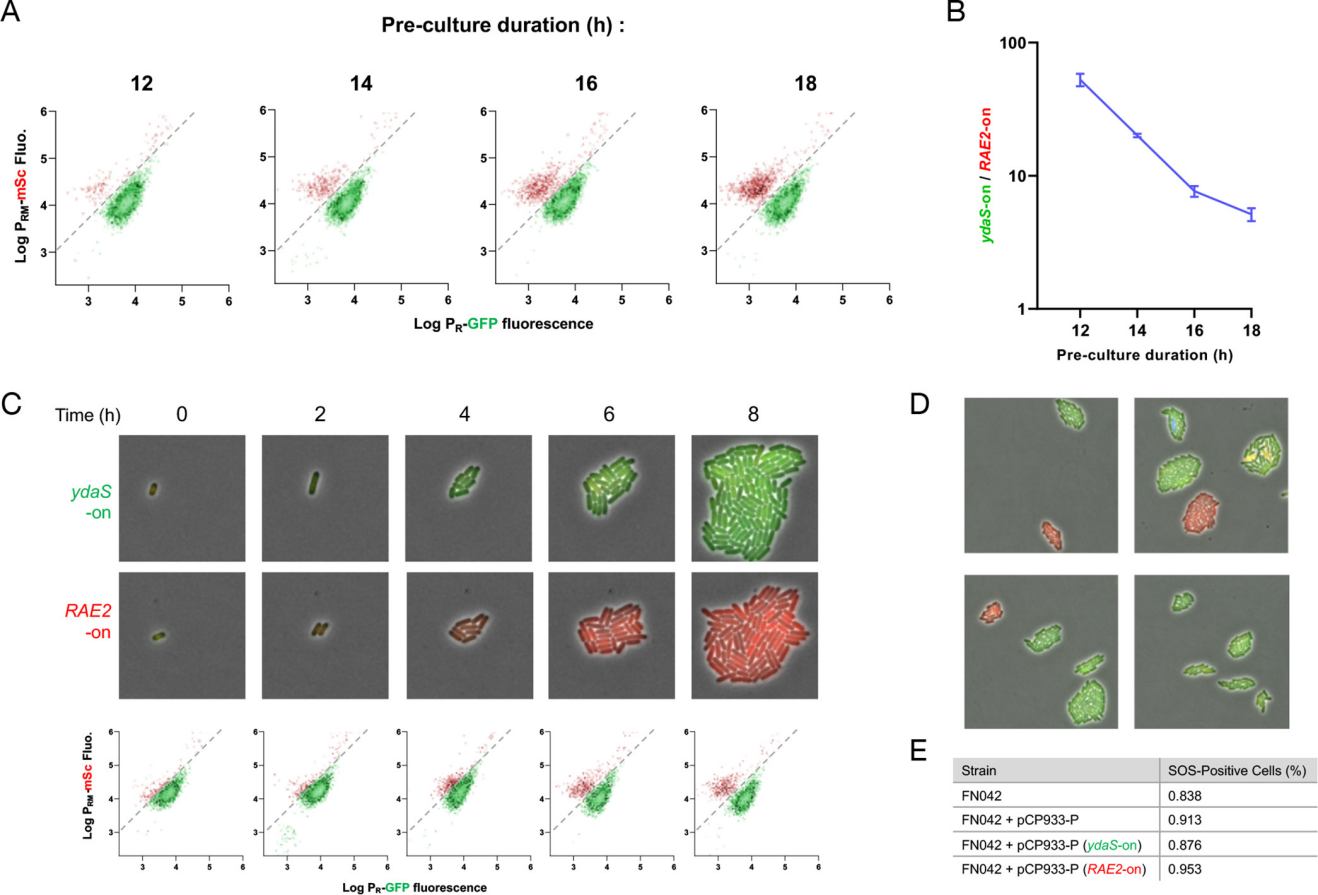
Switching and stable inheritance of the CP-933P immunity transcriptional state. (A) Flow cytometry of exponentially growing cultures inoculated from precultures grown for the indicated duration. (B) Quantification of the *ydaS*-on cells:*RAE2*-on cells ratio for three independent experiments as in panel A. (C) Time-lapse microscopy of FN042 (P*_sulA_-mTagBFP2*) cells transformed with pCP-933P, showing two representative *ydaS*-on and *RAE2*-on microcolonies (top and middle, respectively) and corresponding flow cytometry analysis (bottom). (D) Fluorescence microscopy snapshots of FN042 (P*_sulA_-mTagBFP2*) cells transformed with pCP-933P wt and grown on MOPS glucose agarose pads for 6 h as in panel C. (E) SOS induction in pCP-933P. Cells were grown for 6 h in MOPS glucose medium and analyzed by flow cytometry. The table reports the number of SOS-positive cells (>3,000 arbitrary units [AU] of P*_sulA_-mTagBFP2* fluorescence, as shown in Fig. S4C).

10.1128/mBio.02947-21.7 FIG S4 (A) Flow cytometry background signal values. FN042 cells transformed with pCP-933P nonfluorescent were analyzed by flow cytometry as in Fig. 4B. (B) CP-933P immunity commitment as a function of initial fluorescence. FN042 cells transformed with pCP-933P wild type (wt) were grown and imaged on agarose pads as in Fig. 5. Median cytosolic green fluorescent protein (GFP) and mScarlet-I (mSC) fluorescence of mother cells at time zero were quantified and plotted in green for cells that generated *ydaS*-on microcolonies and in red for cells that generated *RAE2*-on microcolonies. (C) Validation of the *sulA* transcriptional fusion. FN042 (P*_sulA_*-*mTagBFP2*) cells were grown for 5 h in morpholinepropanesulfonic acid (MOPS) glucose medium (dark blue histogram) and treated for 3 h with 5 mg/L ofloxacin (light blue histogram). A threshold of 3,000 arbitrary units (AU) was used for SOS-positive cells. (D) SOS induction in pCP-933P wt subpopulations and corresponding gating. FN042 cells transformed with pCP-933P wt construct were grown in MOPS glucose medium for 6 h and analyzed by flow cytometry. (Left) GFP/mScarlet-I plots, as in Fig. 4B, depicting 41,749 cells. (Middle and right) Daughter plots of the left panel. (Middle) Green gate from the left panel (*ydaS*-on subpopulation). (Right) Red gate from the right panel (*RAE2*-on subpopulation). Download FIG S4, JPG file, 0.5 MB. Copyright © 2021 Jurėnas et al. 2021 Jurėnas et al. https://creativecommons.org/licenses/by/4.0/ This content is distributed under the terms of the Creative Commons Attribution 4.0 International license.

To evaluate the contribution of the PaaR2 and YdaS proteins to the phenotypic heterogeneity observed in the wild-type context, tandem stop mutations were introduced in each of the regulator-encoding genes carried by the reporter plasmid to suppress their expression. Mutations in *paaR2* and/or *ydaS* genes abolished either subpopulation compared to the wild-type context (Fig. 4B). Mutation in *paaR2* resulted in the loss of the minor *RAE2*-on subpopulation, while mutating *ydaS* resulted in the loss of the major *ydaST*-on population (Fig. 4B and C). In the absence of both regulators, the whole population shifted to high GFP and mSc fluorescence, indicative of both *ydaST* and *RAE2* expression (Fig. 4B and C). Since PaaR2 and YdaS compete for the same binding sites (Fig. 2C and D), we suggest that this competition generates bistability, resulting in the two different transcriptional states of the reporter described above.

### Switching and inheritance of the CP-933P immunity transcriptional state.

We then investigated how each subpopulation is formed and what conditions that may favor transition from one state to another. While performing the above-described flow cytometry experiments, we noticed a day-to-day variability in the ratio of *ydaS*-on to *RAE2*-on cells. These experiments were performed on exponentially growing cultures inoculated from precultures, which were themselves inoculated from isolated colonies. We found that preculture duration influenced this ratio, with longer preculture times leading to larger amounts of *RAE2*-on cells (Fig. 5A and B). This suggests that the transition to the *RAE2*-on state requires the stationary phase, with higher probabilities to transition the longer the cells remain in the stationary phase.

To capture the aforementioned switch to the *RAE2*-on state, a kinetic experiment was performed in which cells were analyzed by fluorescence microscopy and flow cytometry for 6 h after the dilution from the stationary-phase overnight culture (Fig. 5C). In the stationary phase (time zero), cells displayed high levels of green fluorescence, indicating an *ydaST*-on state (Fig. 5C). As observed above, a tenth of the population progressively switched to a *RAE2*-on state, these cells progressively diluted the green fluorescence and developed a higher level of red fluorescence that was inherited by the progeny (Fig. 5C). Following the growth of 101 microcolonies, we did not observe switching to another transcriptional state subsequent to the initial differentiation of *RAE2*-on cells, indicating that once the transcriptional state of the CP-933P immunity region is defined during the stationary phase, it remains stable throughout balanced growth (Fig. 5D). Moreover, stationary-phase fluorescence provides a good proxy of microcolony fate, with cells that display low GFP fluorescence showing a higher probability to transition to the *RAE2*-on state, thus supporting the notion that the state in which cells find themselves in the stationary phase influences the switching decision (Fig. S4B).

### Transcriptional activation of the *paaR2-paaA2-parE2* locus does not lead to ParE2 activation.

It is important to note that both subpopulations showed appreciable levels of mScarlet-I fluorescence, indicating that the *paaA2-parE2* TA operon is transcribed regardless of the phenotypic state the cells are committed to (Fig. 4B). We therefore assessed the activity of the ParE2 toxin that induces the SOS response (8) in these two subpopulations by performing flow cytometry experiments with a strain encoding a chromosomal *P_sulA_-mTagBFP2* construct that reports activation of the SOS response (Fig. S4C). The *ydaST*-on subpopulation showed 0.876% SOS-positive cells, while the *RAE2*-on subpopulation showed 0.953% SOS-positive cells. Chi-square analysis of 41,749 cells showed that both subpopulations had no significant differences in their proportions of SOS-positive cells (χ^2^ = 0.2495; *P* = 0.61741) (Fig. 5E and Fig. S4D). Moreover, the whole population shows a proportion of SOS-positive cells (0.913%) similar to that of control cells not transformed with this construct (0.838%; χ^2^ = 1.0556; *P* = 0.304215). These data indicate that transcriptional activation of *paaA2-parE2* does not lead to the activation of the SOS response under steady-state conditions, regardless of the transcriptional state of the CP-933P immunity region. This confirms that transcriptional activation of TA system is not necessarily a proxy for toxin activation (16).

We previously showed that the *RAE2* locus is able to mediate plasmid stabilization by postsegregational killing of plasmid-free cells in a ClpAP- and ClpXP-dependent manner (8). To evaluate the capacity of the entire *ydaST-RAE2* locus to exert the same phenotype, this region was cloned in an unstable mini-F plasmid that constitutively produces the mNeongreen fluorescent protein. Figure S5 in the supplemental material shows that the *ydaST-RAE2* locus stabilizes the mini-F plasmid as efficiently as the *RAE2* locus and the *ccdAB* system, a well-characterized type II TA system (49). These data confirm that the ParE2 toxin is activated upon plasmid loss, regardless of the genetic context (with or without the *ydaST* locus).

10.1128/mBio.02947-21.8 FIG S5 Plasmid stabilization by the *ydaST*-*RAE2* locus. (A) Illustration of the loci cloned in the unstable plasmid. (B) Plasmid stability assay. FN042 cells transformed with pNF06 (mini-F Δ*sopABC proDp-mNeongreen*) were grown without selection for 10 h. Plasmid retention (mNeongreen-positive cells) in the population was then quantified in the population by flow cytometry. Download FIG S5, JPG file, 0.2 MB. Copyright © 2021 Jurėnas et al. 2021 Jurėnas et al. https://creativecommons.org/licenses/by/4.0/ This content is distributed under the terms of the Creative Commons Attribution 4.0 International license.

## DISCUSSION

In contrast to a typical TA module, the *paaR2-paaA2-parE2* locus of the cryptic prophage CP-933P has been characterized as a three-component operon (8). The TA genes are preceded by an ORF encoding the transcriptional regulator PaaR2. The *paaR2* gene is located at the exact position occupied by the *cI* and *dicA* genes in lambda and in the E. coli K-12 Qin cryptic prophage, respectively (see Fig. S1 in the supplemental material). Despite low sequence similarity, we could predict using HHpred (50) that PaaR2, like CI and DicA, contains a helix-turn-helix (HTH) motif that is commonly found in prokaryotic transcriptional regulators (51, 52). These motifs are also frequently detected in type II antitoxins, such as HigA, HipB, MqsA, and PezA (53–56). The PaaA2 antitoxin lacks any detectable DNA-binding motifs (8, 9). Even though it could be imagined that PaaR2 and PaaA2 are “simply” a split antitoxin, the transcriptional control of this TA system appears to be embedded in the regulation of the immunity region of the CP-933P prophage. In addition to the regulation by PaaR2, transcription of the *paaR2-paaA2-parE2* operon is also regulated by YdaS and YdaT, which are encoded on the opposite strand of *paaR2-paaA2-parE2* and are likely to be functional analogs of the Cro and CII lambda regulators. Our data suggest that PaaR2 and YdaS compete for DNA-binding sequences located in the intergenic region between the two loci, while YdaT upregulates transcription from a cryptic promoter embedded between the *ydaS* and *ydaT* genes (Fig. 3E). Overall, these data point toward the hypothesis that the *paaA2-parE2* TA system might have inserted within the immunity region of the CP-933P cryptic prophage. Whether the DNA-binding domain of the putative ancestor of the PaaA2 antitoxin has progressively decayed during evolution to reach a total dependency on other transcriptional regulators remains an interesting evolutionary question. Nevertheless, the *paaR2-paaA2-parE2* and *ydaS-ydaT* loci globally maintain a regulation scheme similar to that of the lambda immunity region. Such regions in lambda-like phages typically dictate the lysogeny/lysis decision. CI is rapidly synthesized during lysogeny establishment and further maintains stable expression, which favors the lysogenic lifestyle (57, 58). As mentioned above, PaaR2 shares similarities with the CI lambda repressor, although it lacks the peptidase motif involved in RecA-mediated autocleavage upon SOS induction (see Fig. S1 in the supplemental material) and is therefore not reactive to mitomycin C treatment (26). It also remains unclear whether YdaT shares further similarities with CII, such as the regulation of its activity by proteolysis and by CIII-like protein, that are not found in the CP-933P cryptic prophage (59).

Our single-cell experiments failed to detect any activity of ParE2, even under conditions in which the *paaA2-parE2* locus is expressed. These data are in agreement with a recent study showing that transcriptional activation of TA operons by multiple stressing conditions does not lead to a detectable phenotype (16), which leaves the question of their role(s) open. Interestingly, the genome of the Sp12 prophage, a homologue of CP-933P in the O157:H7 Sakai strain, was shown to be prone to inversions (60–62). In general, cryptic prophages are malleable and polymorphic, with variations largely due to recombination, duplication, and gene loss or acquisition events (5). Despite that, the genomic region encoding *paaR2-paaA2-parE2* appears to be stable (62), arguing for the idea that this TA system might contribute to the genomic stability of the CP-933P cryptic prophage. For example, the immunity region of lambda has been described to readily recombine with homologous phages (e.g., phages 434 and 21), thus generating heteroimmune phages (63). In this context of recombination, *paaA2-parE2* systems could provide protection against recombination by killing lysogens that inherit an immunity region lacking the TA system. This hypothesis is supported by the confirmation that the *RAE2* locus stabilizes an otherwise unstable plasmid (Fig. S5) (8). Therefore, we propose that this TA is an addiction module leading to stabilization and minimizing genomic rearrangements within the CP-933P cryptic prophage.

## MATERIALS AND METHODS

### Strains and plasmids.

See Table S2 in the supplemental material for strains and plasmids used in this study.

### Cloning procedures.

All primer sequences are given in Table S3. PCRs were performed using Q5 DNA polymerase (NEB). Restriction-ligation cloning reactions were performed using *ad hoc* restriction enzymes (NEB) and T4 DNA polymerase (NEB). All constructs were verified by Sanger sequencing.

10.1128/mBio.02947-21.3 TABLE S3 Sequences of oligonucleotides used in this work. Download Table S3, DOCX file, 0.02 MB. Copyright © 2021 Jurėnas et al. 2021 Jurėnas et al. https://creativecommons.org/licenses/by/4.0/ This content is distributed under the terms of the Creative Commons Attribution 4.0 International license.

In order to detect the translation start of PaaR2, the C-terminal fusion with a hexahistidine tag was cloned into the pBAD24 vector to replace the +1 (transcription start) from the P*_araBAD_* promoter with the +1 of P_RM933P_ detected by 5′ RACE (Fig. 1B and C), using primers F-1-PaaR2 and R-PaaR2-His-SphI and E. coli O157:H7 EDL933 DNA as the template. The vector scaffold was amplified from pBAD24 using primers F-SphI-pBAD and R-ara-prom. These two fragments were digested with SphI, phosphorylated with T4 polynucleotide kinase (PNK; NEB) and ligated to yield pBAD24-PaaR2-His. The pBAD24-P_RM933P_-PaaR2-His plasmid, in which the P*_araBAD_* promoter was replaced with the P_RM933P_ promoter, was constructed as described for pBAD24-PaaR2-His, using primers F-prom-PaaR2 and R-PaaR2his-SphI to amplify PaaR2-His and F-SphI-pBAD and R-pBAD-noprom to amplify the vector scaffold. Expression of the PaaR2-His protein was confirmed by Western blotting with anti-His antibodies (Sigma-Aldrich).

For promoter activity assays, pPROBE′-*gfp* vectors were generated as follows. Fragments containing PRM, PR, PL, or PRE promoter sequences were amplified from EDL933 DNA using the primers F-PRM-SacI/R-PRM BamHI, F-PR-SacI/R-PR BamHI, F-PL-SacI/R-PL BamHI, and F-PRE-SacI/R-PRE BamHI, respectively. Fragments were digested with SacI and BamHI and ligated with pPROBE′-*gfp* vector digested with the same enzymes. Putative regulators and controls were cloned in the same manner in the pBAD24 vector, using EcoRI and HindIII restriction sites.

The pCP-933P vectors were generated as follows. The immunity region spanning promoter P_L933P_, the *parE2*-*paaA2*-*paaR2* locus, the intergenic region with promoters P_RM933P_ and P_R933P_, and the *ydaS*-*ydaT* locus with promoter P_RE933P_ was amplified using primers F-CP933P-Xba and R-CP933P-Sal from EDL933 DNA. A plasmid scaffold comprising the *cat* gene and a p15A origin of replication was amplified from pCNS (a derivative of pSU18) (64) using F-CNS-Xho and R-CNS-Nhe primers. The amplicons were column purified and digested with XbaI and SalI for the insert and XhoI and NheI for the vector, thus providing compatible ends (Nhe-Xba and Xho-Sal) that could be ligated without forming restriction sites. This nonfluorescent pCP-933P plasmid was then used to introduce fluorescent proteins in the 5′ and 3′ ends of the immunity region. The *gfp* gene was amplified from the pPROBE′-*gfp* vector using the primers F-GFP-Sal and R-GFP-Hind and introduced after *ydaST* by amplifying the pCP-933P nonfluorescent vector with F-pCPYda-Hind and R-pCPYda-Sal primers, cleaving both amplicons with HindIII and SalI, followed by ligation. The mScarlet-I gene was amplified from the pNF02 plasmid (65) using the primers F-mSc-Pst and R-mSc-Eco and introduced after *paaR2*-*paaA2*-*parE2* by amplifying the aforementioned vector with F-pCPRAE-Eco and R-pCPRAE-Nsi primers, cleaving the backbone with EcoRI and NsiI and the insert with EcoRI and PstI. Ligation of these fragments yielded the pCP-933P wild-type (wt) plasmid.

Elimination of the expression of each regulator from the pCP-933P wt plasmid was generated by introducing two consecutive ochre stop codons (TAATAA) at the beginning of the regulator genes, at the position of the 10th and 11th amino acids. Mutations to be inserted into *ydaS* and *paaR2* were encoded on the primers F-YdaS-stop/R-YdaS-stop and F-PaaR2-stop/R-PaaR2-stop, respectively. The PCR products amplified from pCP-933P wt using these primers were phosphorylated and ligated to yield pCP-933P-PaaR2^mut^, pCP-933P-YdaS^mut^, and pCP-933P-PaaR2^mut^-YdaS^mut^.

The pNF06 unstable plasmid was constructed by first inserting a synthetic proDp-*mNeongreen* synthetic gene at the SalI and PciI sites of pBeloBAC11. To make the plasmid unstable, the *sopABC* partition complex was deleted with the primers miniF-del-sop for/rev. The *aphA2* kanamycin resistance cassette was then amplified from pKD13 with the primers KmR for/rev and cloned between XhoI and MluI sites, thus replacing the *cat* chloramphenicol resistance cassette and yielding pNF06. The *ccdAB*, *RAE2*, and *ydaST-RAE2* operons were amplified using the primers 06ccd for/rev, 06RAE2 for/rev, and 06STRAE2 for/rev, respectively, and cloned at the AatII and SacI sites of pNF06.

Strain FN042 was constructed by inserting a synthetic P*_sulA_-mTagBFP2* construct based on strain SMR6039 (66) at the BmtI and SalI sites of pKD13. A cassette containing the reporter and an FLP recombination target (FRT)-flanked kanamycin resistance cassette was amplified using the primers sulAbfp for/rev and inserted in the chromosome of strain MG1655 using lambda red recombineering (67). The kanamycin resistance cassette was excised using pCP20. Functionality of the reporter was validated using ofloxacin at 5 μg/mL (Fig. S4C).

### Transcription start site mapping with 5′ RACE.

Total RNA was extracted from E. coli O157:H7 EDL933 at an OD_600_ of 1.5 by extraction with 65°C phenol, followed by chloroform extraction and precipitation with ethanol. RNA was resuspended in RNase-free water, and quality was confirmed by visualizing on agarose gel. A 30-μg aliquot of RNA was treated with 25 units of tobacco acid pyrophosphatase (TAP; Westburg) for 2 h at 37°C. RNA was then reextracted with phenol-chloroform and precipitated with ethanol, resuspended in water, and ligated with RNA adapter (Table S3) using T4 RNA ligase (NEB) overnight at 16°C. The reaction was followed by reextraction and precipitation as described before. A 3-μg aliquot of TAP-treated RNA was used for reverse transcription reaction with SuperScript II reverse transcriptase (Thermo Fisher) with the primers listed in Table S3. A 4-μl aliquot of the reverse transcription (RT_ reaction was then used for PCR using a primer complementary to the RNA adapter and a nested specific primer (Table S3). A 4-μL aliquot of PCR product was then used for TOPO-TA cloning with a kit (Invitrogen), and the reaction product was then transformed into TOP10 electrocompetent cells. The resulting clones were analyzed by PCR with M13 forward/reverse primers, and 5 independent clones for each 5′ RACE experiment were sequenced to map the transcription start site.

### Protein expression and purification.

PaaR2 protein expression and purification were performed as previously described (42). YdaS protein expression and purification were performed as previously described (24).

PaaR2 for translation start detection was purified by affinity chromatography as previously described from a culture of DJ624 Δ*ara* (pBAD24-PaaR2-His) cells, induced with 1% arabinose. The molecular weight of the purified protein was then determined using mass spectrometry (intact mass measurement).

### Measurements of promoter activities and *in vivo* repression.

To test promoter activities, overnight cultures of DJ624 Δ*ara* cells transformed with pPROBE′-Promoter-*gfp* and pBAD24-Regulator vectors were diluted to an OD_600_ of 0.02 in M9 minimal medium supplemented with Casamino Acids (0.2%), 50 μg/mL kanamycin, 100 μg/mL ampicillin, and 0.2% glucose. Cultures (1 mL) were grown in glass-bottomed black 24-well plates (Greiner) in a SpectraMax i3 microplate reader (Molecular Devices) at 37°C with constant shaking. Optical density (600 nm) and fluorescence (excitation, 485 nm; emission, 520 nm) were measured every 15 min. After 3 h of growth, 0.2% arabinose was added to produce the PaaR2, YdaS, and YdaT regulators.

The strain with empty pPROBE′-*gfp* vector cotransformed with each promoter was used as a blank using the formula $I(t)=I_{u}(t)-\frac{A(t)}{B(t)}I_{b}(t)$, as described previously (68). *I*(*t*) is the fluorescence intensity at a specific time point, *I_u_*(*t*) is the uncorrected intensity, *A*(*t*)/*B*(*t*) is the absorbance ratio between the measured culture and the culture used as a blank, and *I_b_*(*t*) is the fluorescence intensity of the culture used as a blank. The blanked values were then divided by OD_600_ values at each time point to obtain corrected fluorescence intensity per OD_600_ unit.

### Electrophoretic mobility shift assays.

In order to characterize protein-DNA binding, electrophoretic mobility shift assays (EMSAs) were performed using PCR-amplified DNA. DNA was prepared as follows. The full-length promoter/operator region and a random intergenic region were generated by colony PCR on E. coli O157:H7 strain EDL933 using primer pairs FL1/FL2 and Neg1/Neg2 (Table S3), respectively. Both fragments have a length of 97 bp and similar GC contents. The PCR products were purified using the Wizard SV gel and PCR clean-up system (Promega) to obtain a concentration of 2.5 μM.

Binding reactions were performed in phosphate-buffered saline (PBS) at pH 8.0, supplemented with 1 mM Tris(2-carboxyethyl)phosphine hydrochloride (TCEP) in the case of YdaS, in a total volume of 10 μl. The final concentrations of DNA were 0.25 μM for the longer fragments and 0.5 μM for the individual boxes. The mixture was incubated for 30 min at 20°C. After addition of 2 μl of loading dye (25% Ficoll 400, 0.1% xylene cyanol, and 0.1% bromophenol blue), the samples were loaded on a native 6% polyacrylamide gel prepared with Tris-borate-EDTA (TBE) buffer (89 mM Tris-HCl, 89 mM boric acid, and 2.5 mM EDTA) that had been prerun for 30 min at 100 V on ice. The electrophoresis was performed for 10 min at 180 V and for 30 to 40 min at 120 V, using TBE as the running buffer. The gel was then stained using ethidium bromide.

### DNase I footprinting.

In order to identify the binding sites of PaaR2 and YdaS, DNase I footprinting experiments were performed according to the method of Galas and Schmitz (69) with minor modifications. The DNA fragment for the full-length promoter/operator region was generated by colony PCR on E. coli O157:H7 strain EDL933 using the primers Footprint1 and Footprint2 (Table S3). One of the primers was 5′-end-labeled with γ-^32^P-ATP (Perkin Elmer) and T4 polynucleotide kinase (Fermentas). The labeled PCR fragment was then purified by electrophoresis on a 6% native polyacrylamide gel. Binding reactions were performed in 20 mM Tris HCl (pH 7.3) and 150 mM NaCl in a total volume of 50 μl for PaaR2 and in PBS (pH 8.0) and 1 mM TCEP for YdaS, over 30 min at 20°C. DNase I (0.2 units; Roche) was then added to each sample, and the reaction was stopped after 2.5 min by adding 12.5 μL of DNase stop mix (3 M ammonium acetate and 0.25 M EDTA) and 15 μg of yeast tRNA. The DNA was then precipitated with ethanol and analyzed on a denaturing 8% polyacrylamide gel. The reference ladders were generated by chemical DNA sequencing (70).

### Flow cytometry.

Cultures grown in morpholinepropanesulfonic acid (MOPS) medium supplemented with 0.4% glucose (referred to as MOPS glucose) were diluted in PBS to an OD_600_ of 0.01 and processed by an Attune NXT flow cytometer (Thermo Fisher) at a flow rate of 12.5 μL/min. Fluorescence pulse heights were acquired using a blue laser (488 nm) and a 522/31 emission filter for GFP, a yellow laser (561 nm) and a 603/48 emission filter for mScarlet-I, and a violet laser (405 nm) and a 440/50 emission filter for mTagBFP2. Doublets were gated out based on the area-to-height ratio of their side-scatter pulses. Photomultiplier tube (PMT) gain was adjusted to obtain background median values of ∼10^3^ arbitrary units (AU) (see Fig. S4A).

### Fluorescence microscopy.

Cultures were spotted on a 5-mm × 5-mm pad of solid medium containing MOPS glucose medium and 2% agarose. The inoculated pad was sealed inside a 17-mm × 28-mm Gene Frame (Thermo Fisher) and imaged under an inverted fluorescence microscope (Axio Observer; Zeiss). Illumination was provided using an HXP 120-V light source at 20% intensity. GFP was imaged using a 38 HE filter (excitation at 470/40 nm and emission at 525/40 nm; Zeiss), mScarlet-I using a custom filter (excitation at 560/40 and emission at 590LP; Chroma), and mTagBFP2 using a custom filter (excitation at 405/20 and emission at 470/24; Chroma). Exposure times were 1,500 ms, 200 ms, and 500 ms, respectively. Time-lapse imaging was performed as described above using a plexiglass enclosure heated at 37°C (Pecon) and by taking images every 15 min. Median cellular fluorescence was measured using MicrobeJ (71).
