# Supplementary figures and images for: Bistable Expression of a Toxin-Antitoxin System Located in a Cryptic Prophage of Escherichia coli O157:H7
Source: mBio. 2021 Nov 30;12(6):e02947-21. doi: 10.1128/mBio.02947-21 (PMC8630535; doi:10.1128/mBio.02947-21)

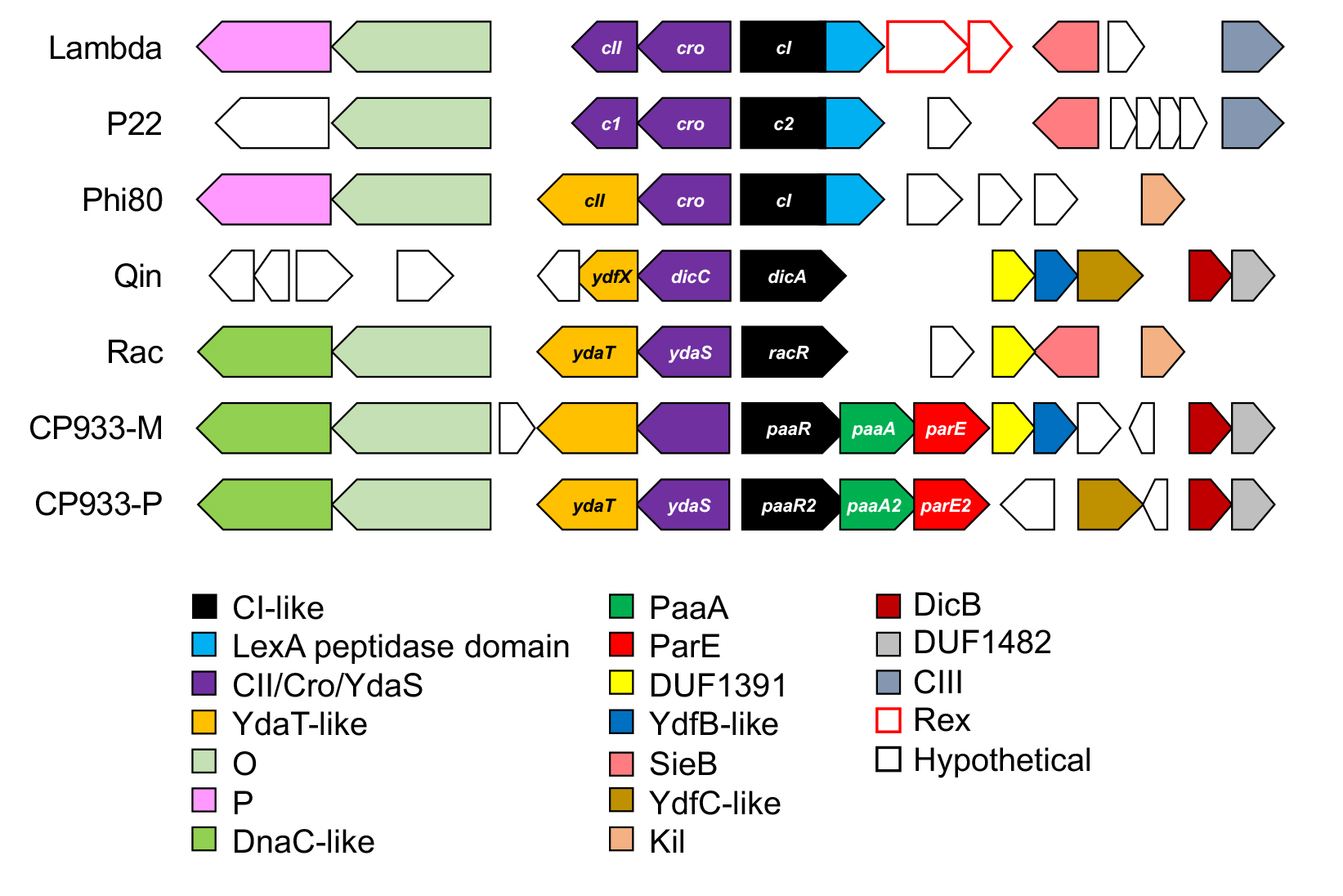

Supplement: FIG S1 [file mbio.02947-21-sf001.jpg]

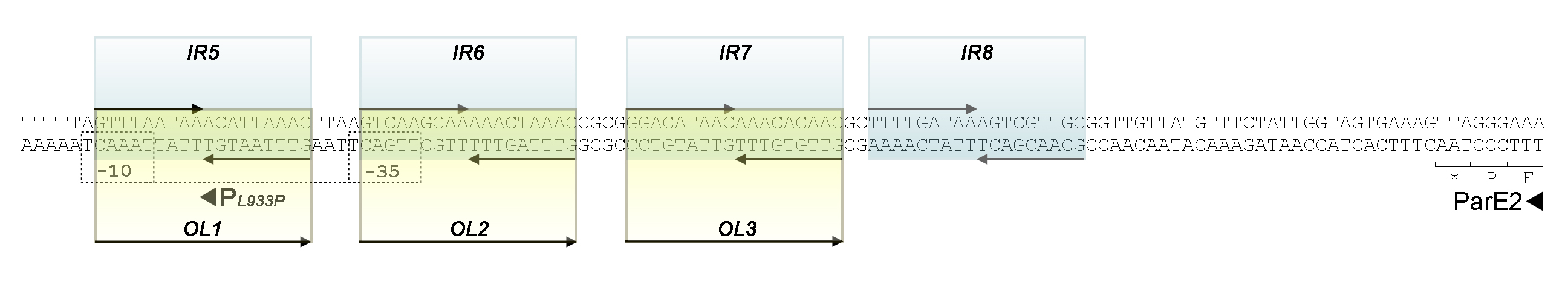

Supplement: FIG S2 [file mbio.02947-21-sf002.jpg]

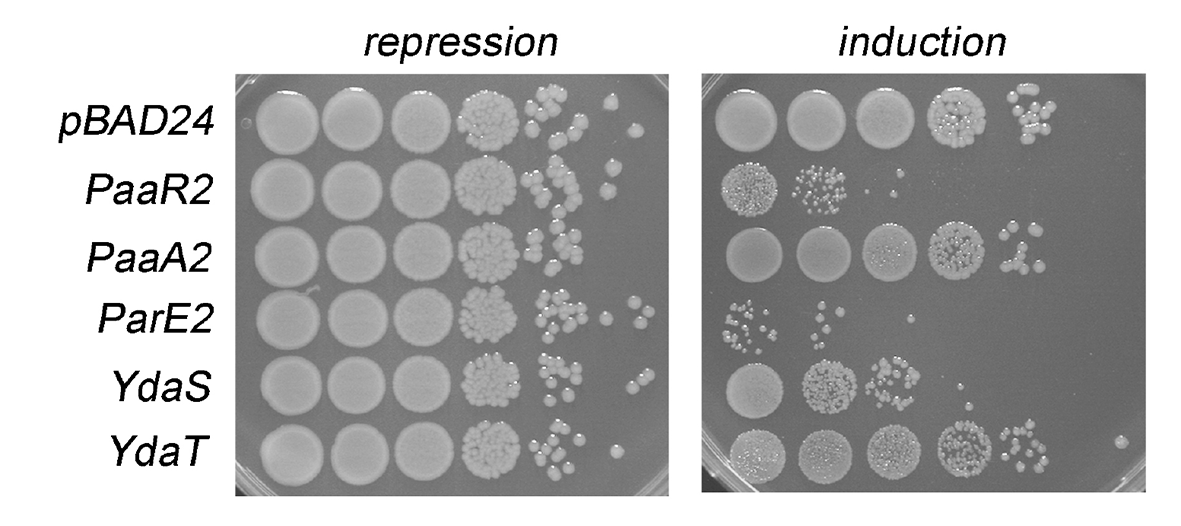

Supplement: FIG S3 [file mbio.02947-21-sf003.tif]

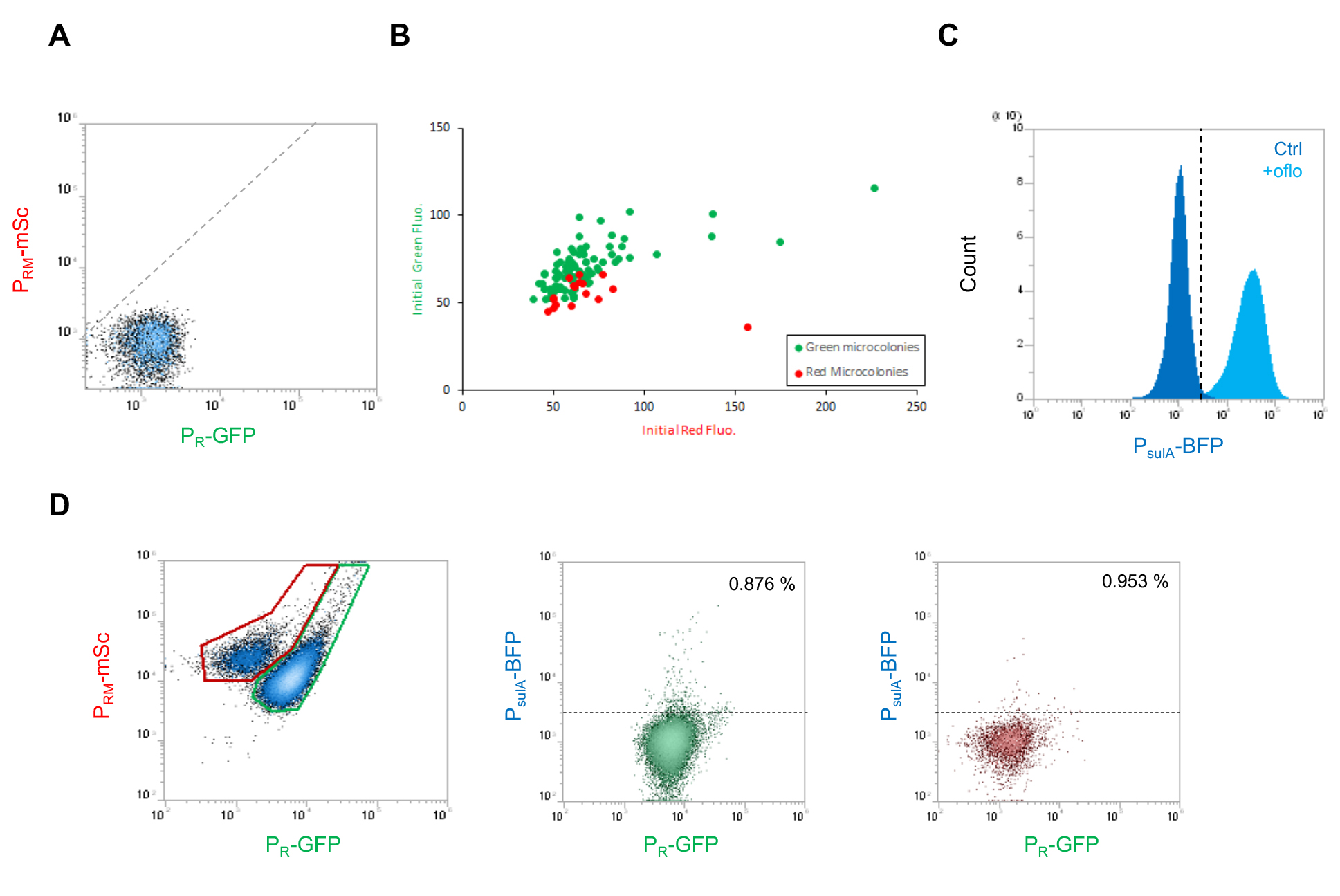

Supplement: FIG S4 [file mbio.02947-21-sf004.jpg]

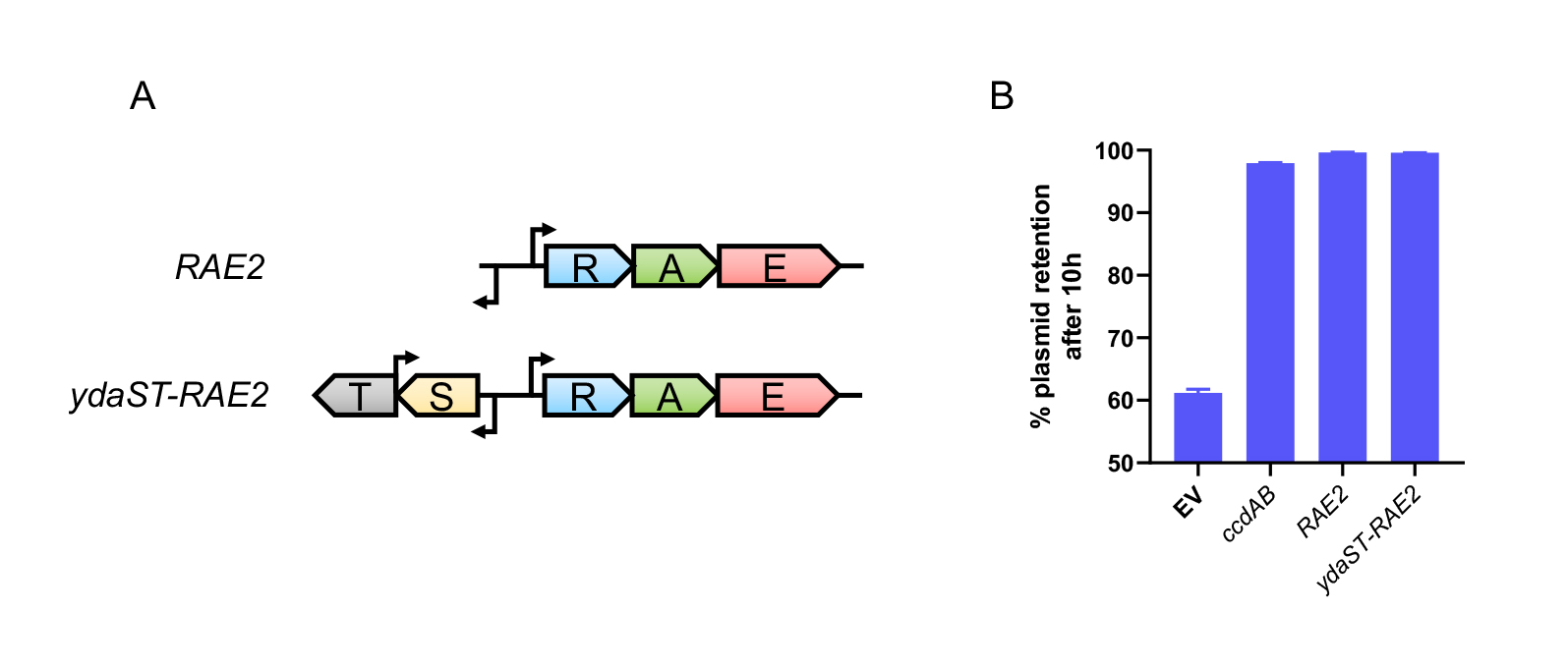

Supplement: FIG S5 [file mbio.02947-21-sf005.jpg]
