# Supplementary material for: Bistable Expression of a Toxin-Antitoxin System Located in a Cryptic Prophage of Escherichia coli O157:H7
Source: mBio. 2021 Nov 30;12(6):e02947-21. doi: 10.1128/mBio.02947-21 (PMC8630535; doi:10.1128/mBio.02947-21)
Supplement: TABLE S1 [file mbio.02947-21-st001.docx]

**Table S1.** Lambda prophage promoters in comparison to CP-933P prophage promoters and consensus promoter sequence (bold)
