# Supplementary material for: Bistable Expression of a Toxin-Antitoxin System Located in a Cryptic Prophage of Escherichia coli O157:H7
Source: mBio. 2021 Nov 30;12(6):e02947-21. doi: 10.1128/mBio.02947-21 (PMC8630535; doi:10.1128/mBio.02947-21)
Supplement: TABLE S2 [file mbio.02947-21-st002.docx]

**Table S2.** Strains and plasmids used in this work

| **Strains** | **Genotype or description** | **Source** |
| --- | --- | --- |
| MG1655 | *Escherichia coli* K-12 *rph-1* *rfb-1* *ilvG* | Lab collection |
| DJ624Δ*ara* | MG1655 *lacX74 malP::lacI_q_* Δ*ara* | (1) |
| O157:H7 EDL933 | Enterohemorrhagic *E. coli* | (2) |
| FN042 | MG1655 *fhuA::*P*_sulA_-mTagBFP2-FRT* | This study |
| **Plasmids** |  |  |
| pBAD24 | pBR322 *ori*, *bla*, P*_araBAD_* promoter | (3) |
| pBAD24-PaaR2-His | pBAD24, *paaR2*-his cloned at +1 of *araB* | This study |
| pBAD24-P_RM933P_-PaaR2-His | pBAD24, P*_araBAD_* promoter replaced by P_RM933_-*paaR2*-*His* | This study |
| pBAD24-PaaR2 | pBAD24, *paaR2_EDL933_* cloned after P*_araBAD_* and RBS | This study |
| pBAD24-YdaS | pBAD24, *ydaS* *_EDL933_* cloned after P*_araBAD_* and RBS | This study |
| pBAD24-YdaT | pBAD24, *ydaT* *_EDL933_* cloned after P*_araBAD_* and RBS | This study |
| pPROBE'-*gfp* | pBBR1 *ori*, *aphA2*, *gfp*[tagless] | (4) |
| P_R933P_-*gfp* | pPROBE', P_R933P_ cloned upstream of *gfp* | This study |
| P_RM933P_-*gfp* | pPROBE', P_RM933P_ cloned upstream of *gfp* | This study |
| P_L933P_-*gfp* | pPROBE', P_L933P_ cloned upstream of *gfp* | This study |
| P_RE933P_-*gfp* | pPROBE', P_RE933P_ cloned upstream of *gfp* | This study |
| pCP-933P non-fluo | P15A *ori*, *cat*, immunity of CP933P comprising P_L933P_-*parE2-paaA2-paaR2*-P_RM933P_-P_R933P_-*ydaS-ydaT* | This study |
| pCP-933P wt | pCP-933P non-fluo backbone, *mScarlet-I* inserted after *parE2*, *gfp* inserted after *ydaST*. | This study |
| pCP-933P-PaaR2^mut^ | pCP-933P wt, stop codon mutations introduced in *paaR2* | This study |
| pCP-933P-YdaS^mut^ | pCP-933P wt, stop codon mutations introduced in *ydaS* | This study |
| pCP-933P-PaaR2^mut^-YdaS^mut^ | pCP-933P wt, stop codon mutations introduced in *paaR2* and *ydaS* | This study |
| pKD13 | R6Kori *FRT-aphA2-FRT* *bla* | (5) |
| pCP20 | *cI857* P_Lλ_*-flp* *bla* *cat* | (5) |
| pBeloBAC11 | mini F *sopABC^+^ cat lacZα cos_λ_* | NEB |
| pNF06 | mini F Δ*sopABC aph-2 proDp-mNeongreen* | This study |
| pNF06-*ccdAB* | pNF06 *ccdAB* | This study |
| pNF06-*RAE2* | pNF06 *paaR2-paaA2-parE2* | This study |
| pNF06-*ydaST-RAE2* | pNF06 *ydaS-ydaT-paaR2-paaA2-parE2* | This study |

1. Hallez R, Geeraerts D, Sterckx Y, Mine N, Loris R, Van Melderen L. 2010. New toxins homologous to ParE belonging to three-component toxin-antitoxin systems in Escherichia coli O157:H7. 3. Mol Microbiol 76:719–732.

2. Riley LW, Remis RS, Helgerson SD, McGee HB, Wells JG, Davis BR, Hebert RJ, Olcott ES, Johnson LM, Hargrett NT, Blake PA, Cohen ML. 1983. Hemorrhagic colitis associated with a rare Escherichia coli serotype. N Engl J Med 308:681–685.

3. Guzman LM, Belin D, Carson MJ, Beckwith J. 1995. Tight regulation, modulation, and high-level expression by vectors containing the arabinose PBAD promoter. J Bacteriol 177:4121–4130.

4. Miller WG, Leveau JH, Lindow SE. 2000. Improved gfp and inaZ broad-host-range promoter-probe vectors. Mol Plant Microbe Interact 13:1243–1250.

5. Datsenko KA, Wanner BL. 2000. One-step inactivation of chromosomal genes in Escherichia coli K-12 using PCR products. Proc Natl Acad Sci U S A 97:6640–6645.
