# Supplementary material for: Bistable Expression of a Toxin-Antitoxin System Located in a Cryptic Prophage of Escherichia coli O157:H7
Source: mBio. 2021 Nov 30;12(6):e02947-21. doi: 10.1128/mBio.02947-21 (PMC8630535; doi:10.1128/mBio.02947-21)
Supplement: TABLE S3 [file mbio.02947-21-st003.docx]

**Table S3.** Sequences of oligonucleotides used in this work.

| **primer name** | **primer sequence 5’-3’** | **Purpose** |
| --- | --- | --- |
| RNA adapter | ACUCUCUACUGUUUCUCCAU | RNA adapter for 5’RACE |
| PaaR2 RT1 | GCGCCGCAATCCATCTCGCT | Reverse transcription for 5'RACE |
| YdaS RT1 | CGCCATGCGGATGTGTTCATGCTTG | Reverse transcription for 5'RACE |
| anti-adapter | ACCTGACGCTTTTTATCGCAACTCTCTACTGTTTCTCCAT | PCR for 5'RACE |
| PaaR2 RT2 | CGTTGCTCATCCTCAGGGAAGGCT | PCR for 5'RACE |
| YdaS RT2 | CCCAATCATGGGGTCGTAGCTCCG | PCR for 5'RACE |
| F-1-PaaR2 | ATGCAAAAAAAAGAAATTCGCCGTTTAC | Construction of pBAD24-PaaR2-His |
| R-PaaR2his-SphI | GATCGCATGCTCAGTGGTGGTGGTGGTGGTGGGC  GCGGCGGCATTTTTGC | Construction of pBAD24-PaaR2-His and pBAD24-P_RM933P_-PaaR2-His |
| F-SphI-pBAD | CCCCGCATGCAAGCTTGGCTGTT | Construction of pBAD24-PaaR2-His  and pBAD24-P_RM933P_-PaaR2-His |
| R-ara-prom | ATGGAGAAACAGTAGAGAGTTGCG | Construction of pBAD24-PaaR2-His |
| F-prom-PaaR2 | ACCTTCCTCGGTTTAGTGTTTTTTTG | Construction of pBAD24-P_RM933P_-PaaR2-His |
| R-pBAD-noprom | GGTCCCGCTTTGTTACAGAATGC | Construction of pBAD24-P_RM933P_-PaaR2-His |
| F-PRM-SacI | GATCGAGCTCTGCGAGTTTAGTGTTTAATAA | Amplify P_RM933P_ promoter to clone in pPROBE'-*gfp* |
| R-PRM-BamHI | AGTCGGATCCTGTGGTAATTATCTTTAGTAATCACT | Amplify P_RM933P_ promoter to clone in pPROBE'-*gfp* |
| F-PR-SacI | GATCGAGCTCTGTGGTAATTATCTTTAGTAATCACT | Amplify P_R933P_ promoter to clone in pPROBE'-*gfp* |
| R-PR-BamHI | AGTCGGATCCTGCGAGTTTAGTGTTTAATAA | Amplify P_R933P_ promoter to clone in pPROBE'-*gfp* |
| F-PL-SacI | GATCGAGCTCCTTTCACTACCAATAGAAACA | Amplify P_L933P_ promoter to clone in pPROBE'-*gfp* |
| R-PL-BamHI | GATCGGATCCATTTTTAGTTTAATAAACATTA | Amplify P_L933P_ promoter to clone in pPROBE'-*gfp* |
| F-PRE-SacI | AGTCGAGCTCATGCGGATGTGTTCATGCTT | Amplify P_RE933P_ promoter to clone in pPROBE'-*gfp* |
| R-PRE-BamHI | CTGATGGATCCCTGAGTATGCGAGCGGCAT | Amplify P_RE933P_ promoter to clone in pPROBE'-*gfp* |
| F-PaaR2-Eco | GATCGAATTCATGCAAAAAAAAGAAATTCGCCGT | Amplify *paaR2* gene to clone in pBAD24 |
| R-PaaR2-Hind | GATCAAGCCTTCAGGCGCGGCGGCATTT | Amplify *paaR2* gene to clone in pBAD24 |
| F-YdaS-EcoRI | GATCGAATTCATGACATTAAAAGAGTTTATTAAATC | Amplify *ydaS* gene to clone in pBAD24 |
| R-YdaS-HindIII | GATCAAGCTTTCATGAATCAACTCCATCAG | amplify *ydaS* gene to clone to pBAD24 |
| F-YdaT-EcoRI | GATCGAATTCATGAAAATCAAGCATGAACAC | Amplify *ydaT* gene to clone in pBAD24 |
| R-YdaT-HindIII | GATCAAGCTTTTAGTGCATCACCACAGC | Amplify *ydaT* gene to clone in pBAD24 |
| F-CNS-Xho | GGGGCTCGAGGAATGGCGCTGATGTCCGG | Amplify plasmid scaffold for pCP-933P non-fluo |
| R-CNS-Nhe | GGGCGCTAGCGGAGTGTAT | Amplify plasmid scaffold for pCP9-33P non-fluo |
| F-CP933P-Xba | GGGGTCTAGAAAAACCCCCGAACACCGTGCTTTTA | Amplify immunity region for pCP-933P non-fluo |
| R-CP933P-Sal | GGGGGTCGACTGAGAAACCCTCTGTTTCCCCTTAA | Amplify immunity region for pCP-933P non-fluo |
| F-GFP-Sal | GGGGGTCGACAGGAGGGAGTAATGAGTAAAGGAGAAGAACTTTTCAC | Amplify GFP to clone in pCP-933P |
| R-GFP-Hind | GGGGAAGCTTTTATTTGTATAGTTCATCCATGCC | Amplify GFP to clone in pCP-933P |
| F-pCPYda-Hind | GGGGAAGCTTAAGCACGGTGTTCGGGGGTT | Amplify pCP-933P for GFP insertion |
| R-pCPYda-Sal | GGGGGTCGACTTAGTGCATCACCACAGCATTCC | Amplify pCP-933P for GFP insertion |
| F-mSc-Pst | CCCGCTGCAGATTCAGGGAGACCACAACGG | Amplify mScarlet-I in clone to pCP-933P |
| R-mSc-Eco | CCCCGAATTCAGTGTGATACTAGAGGTGCAC | Amplify mScarlet-I in clone to pCP-933P |
| F-pCPRAE-Eco | GGGGGAATTCACTACCAATAGAAACATAACAACCGCAACGAC | Amplify pCP-933P for mScarlet-I insertion |
| R-pCPRAE-Nsi | GGGGATGCATTTAGGGAAACTGGCGTCTTGCG | Amplify pCP-933P for mScarlet-I insertion |
| F-YdaS-stop | TAATAAGGTGATGCTAAGAAATTCGC | Insert stop codons in *ydaS* from pCP-933P |
| R-YdaS-stop | TAATGATTTAATAAACTCTTTTAATGTCA | Insert stop codons in *ydaS* from pCP-933P |
| F-PaaR2-stop | TAATAAGAGTGGTTTAAAGATAAAACTCTGC | Insert stop codons in *paaR2* from pCP-933P |
| R-PaaR2-stop | ACGTAAACGGCGAATTTCTTT | Insert stop codons in *paaR2* from pCP-933P |
| sulA-bfp-for | AGCCAACTTGTGAAATGGGCACGGAAATCCGTGCCCCAAAAGA  GAAATTACTGTAACAGAGCATTAGCGC | Chromosomal insertion of P*_sulA_-mTagBFP2* |
| sulA-bfp-rev | ATAATCATTCTCGTTTACGTTATCATTCACTTTACATCAGAGA  TATACCATCTTGAGCGATTGTGTAGGC | Chromosomal insertion of *P_sulA_-mTagBFP2* |
| miniF-del-sop for | CTAGTTAATTAATCGATGAGCTCCAGGTAGAGGTACAC | Deletion of *sopABC* from pBeloBAC11 |
| miniF-del-sop rev | TGGATCCATATGACGTCGACATGTAAATAATAAAAAAGCCGG | Deletion of *sopABC* from pBeloBAC11 |
| KmR for | CCCGCTCGAGACTGGGCGGTTTTATGG | Replace CmR by KmR in pNF06 |
| KmR rev | CCCCACGCGTAGATCCCCTTAttAGAAGAACTCGTC | Replace CmR by KmR in pNF06 |
| 06ccd for | CCCCGAGCTCGGCTTACTAAAAGCCAGATAACAGTATGCG | Clone *ccdAB* in pNF06 |
| 06ccd rev | CCCCGACGTCTTATATTCCCCAGAACATCAGGTTAATGGCG | Clone *ccdAB* in pNF06 |
| 06RAE2 for | CCCCGAGCTCGTCATACCTTCCTCGGTTTAGTG | Clone *RAE2* in pNF06 |
| 06RAE2 rev | CCCCGACGTCTTAGGGAAACTGGCGTCTTG | Clone *RAE2* in pNF06 |
| 06STRAE2 for | CCCCGAGCTCTTAGTGCATCACCACAGCATTCC | Clone yda*ST-RAE2* in pNF06 |
| 06STRAE2 rev | CCCCGACGTCTTAGGGAAACTGGCGTCTTG | Clone yda*ST-RAE2* in pNF06 |
| FL1 | ACCTTCCTCGGTTTAGTGTT | Amplify DNA for EMSA |
| FL2 | GTGGTAATTATCTTTAGTAATC | Amplify DNA for EMSA |
| Neg1 | GATGAATACCTTGACTGCGA | Amplify DNA for EMSA |
| Neg2 | GCTTTTGCGCAGAAATTTCG | Amplify DNA for EMSA |
| Footprint1 | ATCACCAACCCTTAATGATTTAATA | Amplify DNA for footprint |
| Footprint2 | TCTTTAAACCACTCCTTGAGACG | Amplify DNA for footprint |
